# Supplementary material for: Biological Characteristics of Feline Calicivirus Epidemic Strains in China and Screening of Broad-Spectrum Protective Vaccine Strains
Source: Vaccines (Basel). 2023 Dec 15;11(12):1858. doi: 10.3390/vaccines11121858 (PMC10747013; doi:10.3390/vaccines11121858)
Supplement: Supplementary file 1 [file vaccines-11-01858-s001.zip › vaccines-2701332-supplementary.pdf]

**Table S1.** Neutralizing antibody titers and *in vitro* cross-neutralizating antibody titers of cat sera after immunization with each FCV vaccine strain ((FCV-HB7, FCV-HB10, FCV-HB7&FCV-HB10, FCV-255).

| Group | FCV-HB7 | FCV-HB10 | FCV-FJ1 | FCV-AH3 | FCV-JL18 | FCV-SH192 |
|---|---|---|---|---|---|---|
| FCV-HB7&HB10 | 10.63 | 10.47 | 9.64 | 8.47 | 9.30 | 7.20 |
| FCV-HB7 | 10.47 | 5.46 | 10.30 | 7.20 | 8.47 | 5.00 |
| FCV-HB10 | 5.64 | 10.30 | 8.20 | 5.29 | 9.47 | 7.00 |
| FCV-255 | 1.00 | 2.00 | 2.00 | 1.00 | 5.00 | 1.00 |

Note: neutralizing antibody titers are expressed as 1:log2 value.
